# Factors associated with HIV infection among female sex workers in Brazil

**DOI:** 10.1097/MD.0000000000009013

**Published:** 2018-05-25

**Authors:** Célia Landmann Szwarcwald, Giseli Nogueira Damacena, Paulo Roberto Borges de Souza-Júnior, Mark Drew Crosland Guimarães, Wanessa da Silva de Almeida, Arthur Pate de Souza Ferreira, Orlando da Costa Ferreira-Júnior, Inês Dourado

**Affiliations:** a Health Information Laboratory, Institute of Communication and Scientific and Technological Information in Health, Oswaldo Cruz Foundation, Rio de Janeiro; b Federal University of Minas Gerais, Belo Horizonte, Minas Gerais; c Institute of Biology, Federal University of Rio de Janeiro, Rio de Janeiro; d Collective Health Institute, Federal University of Bahia, Salvador, Bahia, Brazil.

**Keywords:** Brazil, female sex workers, HIV infection, homophily, respondent-driven sampling, risk factors

## Abstract

**Background:**

Female sex workers (FSWs) are one of the most-at-risk population groups for human immunodeficiency virus (HIV) infection. This paper aims at identifying the main predictors of HIV infection among FSW recruited in the 2nd Biological and Behavioral Surveillance Survey in 12 Brazilian cities in 2016.

**Method:**

Data were collected on 4245 FSW recruited by respondent driven sampling (RDS). Weights were inversely proportional to participants’ network sizes. To establish the correlates of HIV infection, we used logistic regression models taking into account the dependence of observations resultant from the recruitment chains. The analysis included socio-demographic sex work characteristics, sexual behavior, history of violence, alcohol and drug use, utilization of health services, and occurrence of other sexually transmitted infections (STIs).

**Results:**

HIV prevalence was estimated as 5.3% (4.4%–6.2%). The odds ratio (OR) of an HIV-positive recruiter choosing an HIV-positive participant was 3.9 times higher than that of an HIV-negative recruiter (*P* < .001). Regarding socio-demographic and sex work characteristics, low educational level, street as the main work venue, low price per sexual encounter, and longer exposure time as a sex worker were found to be associated with HIV infection, even after controlling for the homophily effect. The OR of being HIV infected among FSW who had been exposed to sexual violence at least once in a lifetime (OR = 1.5, *P* = .028) and the use of illicit drugs at least once a week were highly significant as well, particularly for frequent crack use (OR = 3.6, *P* < .001). Among the sexual behavior indicators, not using condoms in some circumstances were significantly associated with HIV infection (OR = 1.8, *P* = .016). Regarding the occurrence of other STI, the odds of being HIV infected was significantly higher among FSW with a reactive treponemal test for syphilis (OR = 4.6, *P* < .001).

**Conclusions:**

The main factors associated with HIV infection identified in our study characterize a specific type of street-based sex work in Brazil and provided valuable information for developing interventions. However, there is a further need of addressing social and contextual factors, including illicit drug use, violence, exploitation, as well as stigma and discrimination, which can influence sexual behavior.

## 1 Introduction

Since the beginning of the acquired immune deficiency syndrome (AIDS) epidemic, female sex workers (FSWs) have been nationally and internationally recognized as a population at high risk for acquiring human immunodeficiency virus (HIV) infection.^[1–6]^

Worldwide studies point to the high levels of HIV prevalence among FSWs compared to the general population. In African countries, there is emerging data showing that FSW carry a disproportionate burden of HIV even in generalized epidemics.^[7,8]^ Studies in Asia^[9–11]^ as well as in developed countries also report higher rates among FSW.^[12,13]^ A systematic review of HIV prevalence studies among key populations in Latin America and the Caribbean estimated a median HIV prevalence among FSW of 2.6%,^[14]^ while the estimated prevalence in the adult population was 0.5%.^[15]^ In Brazil, a study carried out in 2000 to 2001 in some capital cities estimated a prevalence of 6.1% among 2712 FSW,^[2]^ a rate of about 15 times higher when compared with that of the Brazilian female population aged 15 to 49 years.^[16]^

FSWs are considered a high-risk group for acquiring HIV infection^[3,17]^ due to their social vulnerability and factors associated with their work such as multiple sex partners, inconsistent condom use, or coinfection with other sexually transmitted infections (STIs).^[18]^ Studies show that HIV infection is associated with socio-demographic and commercial sex work characteristics,^[19–23]^ such as age and schooling, time span of sex work, place of work, price of commercial sex, and use of drugs,^[24–26]^ which, in turn, is associated with unprotected sex.^[27]^ Findings from a snowball survey carried out in Santos, São Paulo, showed that the use of illicit drugs, especially crack, was one of the main factors associated with HIV infection.^[4]^ Furthermore, structural issues such as stigma and discrimination act as important barriers and hinder access to and use of health services.^[28,29]^

The burden of HIV, syphilis, and other STI urged researchers to conduct studies in Brazil among FSW.^[30–32]^ Furthermore, in Brazil concentrated HIV epidemic,^[33]^ small interventions in this vulnerable group can significantly decrease HIV incidence in the general population.^[34]^ Thus, monitoring factors associated with HIV infection is important not only to support interventions focused on this population group, but also to reduce the spread of HIV infection among clients of FSW, which constitute a bridge population for STI/HIV transmission into the Brazilian population.^[35]^

In general, Brazilian studies conducted among FSW until the mid-2000s used convenience samples, making it difficult to estimate parameters for monitoring the HIV/AIDS epidemic in this population group at the national level.^[36]^ In 2009, an HIV biological and behavioral surveillance survey (BBSS) carried out in 10 Brazilian cities was the first study to use a probabilistic sampling method – respondent driven sampling – (RDS) for the recruitment of FSW.^[37]^ For the analysis of data collected by RDS, a statistical method has been proposed for the estimation of HIV prevalence and its variance, taking into account the dependence of observations resultant from the recruitment pattern.^[38]^ This approach was extended to other statistical analyzes, such as measures of association and multivariate models.^[39]^

In 2016, a 2nd HIV BBSS among FSW was carried out in 12 Brazilian cities, aiming at monitoring STI and risky practices among FSW. Based on improvements in data analysis techniques,^[39]^ the aim of this study was to identify factors associated with HIV infection using logistic regression models.

## 2 Methods

This study is part of the 2nd BBSS, a cross-sectional RDS survey among 4328 FSW collected in 12 Brazilian cities from July to November 2016. The BBSS was designed to estimate the prevalence of HIV, syphilis, and hepatitis B and C and to evaluate knowledge, attitudes, and practices related to HIV infection and other STIs among FSW. The research project was approved by the Ethics Committee of the Oswaldo Cruz Foundation (Protocol 1.338.989).

**Figure 1:**
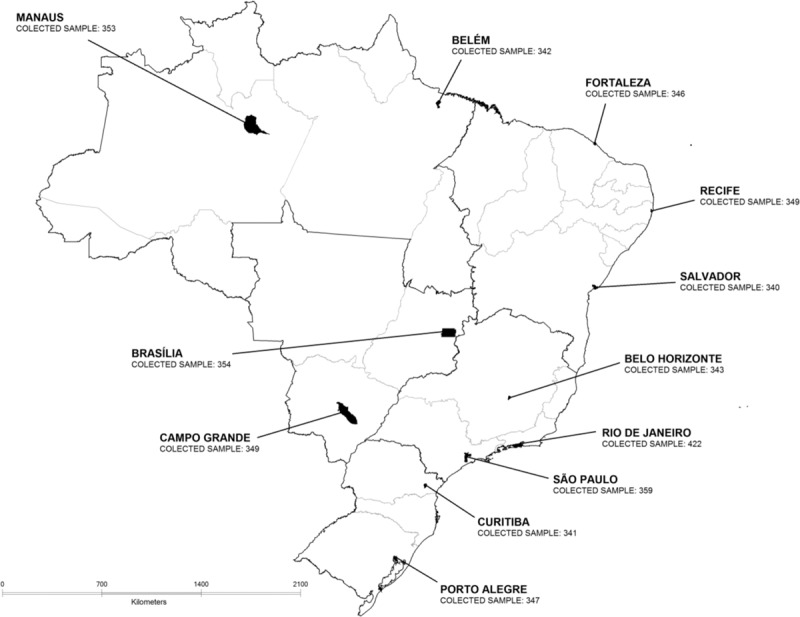
States and the 12 cities where BBSS among FSW was conducted, Brazil, 2016. BBSS = biological and behavioral surveillance survey, FSW = female sex worker.

Twelve Brazilian cities were a priori chosen by the Department of STI/AIDS and Viral Hepatitis, Ministry of Health, according to both, geographical criteria and their epidemiologic relevance in the HIV/AIDS epidemic in the country. The sample size was set at 350 FSW in each city. Figure 1 shows the 12 cities considered in the study and their correspondent sample sizes.

Women were eligible to participate in the study if they met the following inclusion criteria: age 18 years old or over; to report working as a sex worker in one of the cities of the study; to have had at least one sexual intercourse in exchange for money in the past four months; and to present a valid coupon to participate.

Fieldwork was conducted in health services located in the 12 cities. For each city, 6 to 8 initial participants, herein referred to as “seeds,” were chosen purposively, following previous formative research. Seeds were well-connected FSW in their community who reported large social networks. To provide diversity of recruited FSW, seeds were chosen with different characteristics (age group, color/race, socioeconomic class, education, and work venue). Each seed received 3 coupons to distribute to other sex workers from her social network. Recruits of the seeds in the survey were considered the first wave of the study. After participating in the interview, each participant received 3 additional coupons to distribute to their peers and this process was repeated until the sample size was achieved in each city.

The RDS method also draws on the strategy of giving incentives to the participants. A 1st incentive, that is, primary incentive, is given to participants when they complete their participation in the study. Thereafter, a 2nd incentive, that is, secondary incentive, is given to participants for each peer successfully recruited into the study. In this study, the primary incentive was a gift (makeup products), payment for lunch, and transportation in addition to a reimbursement for their time lost from work (approximately US$15.00). The secondary incentive was a payment of US$10.00 for each recruited person who participated in the study. The choice of sites, in general a health service, for data collection and the level of incentives were established according to the formative research carried out in each city before the RDS survey.

The questionnaire included modules on: socio-demographic characteristics and information related to commercial sex activity, knowledge about HIV and other STI transmission, sexual behavior, history of HIV and syphilis testing, STI history, use of alcohol and illicit drugs, access to prevention activities, access to and utilization of health services, discrimination, and violence. The questionnaire was designed for tablets and could be self-administered according to the participant's desire and readiness.

Tests for HIV, syphilis, and hepatitis B and C were conducted by standard rapid tests using peripheral venous blood collection, according to protocols recommended by the Brazilian Ministry of Health. All tests occurred before the interview and all participants received pre- and posttest counseling. Participants who tested positive for any of the rapid tests had their blood samples taken for confirmatory laboratory testing and received additional posttest counseling, both for psychological impact and to encourage partner notification, and were referred to public health systems for follow-up.

Screening for HIV, hepatitis B virus (HBV), hepatitis C virus (HCV), and syphilis antibodies used the following assays: HIV (HIV Test Bioeasy, Standard Diagnostic Inc, Korea and ABON HIV 1/2/O Tri-Line Human Immunodeficiency Virus Rapid Test Device, China), HBV (Vikia HBsAg, BioMérieux SA, France), HCV (ALERE HCV, Standard Diagnostic Inc, Korea), and syphilis, treponemal assay (SD BIOLINE Syphilis 3.0, Standard Diagnostic Inc, Korea). A reactive result on the initial HIV rapid test was followed by a 2nd HIV rapid test, from a different manufacturer and samples reactive on rapid tests were further submitted to confirmatory assays.

### 2.1 Data analysis

The proposed weighting for data collected by RDS is proportional to the inverse of network size of each participant.^[40]^ In this study, the question used to measure the network size of each participant was: “How many sex workers who work here in this city do you know personally?” Each one of the 12 cities composed a stratum and, in each one, the weighting was inversely proportional to the size of the network totaling the size of the stratum.

The tendency of a participant to recruit peers with similar characteristics is usually referred to as homophily.^[41]^ To take into account this bias in the recruitment pattern and a potential overrepresentation of individuals with certain characteristics in the study population, we used logistic regression models to estimate factors associated with HIV infection according to a method proposed by Szwarcwald et al.^[38]^ For each participant, the result of the recruiter's HIV test was taken into account to control for the homophily effect. Additionally, the logistic regression models were performed by taking into account the complex sample design, by considering each city as a stratum and the participants recruited by the same FSW as a cluster.^[38]^

The following variables were included in the analysis: socio-demographic variables (age, educational level, and race/color); characteristics related to sex work (workplace, time as FSW, and price of each sexual encounter); prevention activities (affiliated to/or participated in a non-governmental organization [NGO] to FSW rights [FSW-NGO], STI counseling); sexual behavior (not using condoms in some circumstances – knows the client, in much need of money, client's requirement, not having condom available at the time of the sexual encounter, other); consistent condom use with clients in vaginal sex); history of physical and sexual violence; alcohol and drug use (unprotected sex due to alcohol or drug use at least once a week, crack or cocaine use at least once a week); utilization of health services (Pap smear and HIV testing in the previous 24 months before the survey); and STI (self-referred occurrence of lesions, blisters or warts on the vagina or anus in the previous 12 months, and reactive treponemal antibody test for syphilis).

## 3 Results

HIV prevalence was estimated as 5.3% with a 95% confidence interval (CI) (4.4%–6.2%). The odds ratio (OR) of an HIV-positive recruiter choosing an HIV-positive participant was nearly 4 times higher than that of an HIV-negative recruiter (Table 1). Taking into account the homophily effect and the dependence between recruiters and their recruited participants, the design effect was estimated at 1.76.^[38]^

**Table 1:**
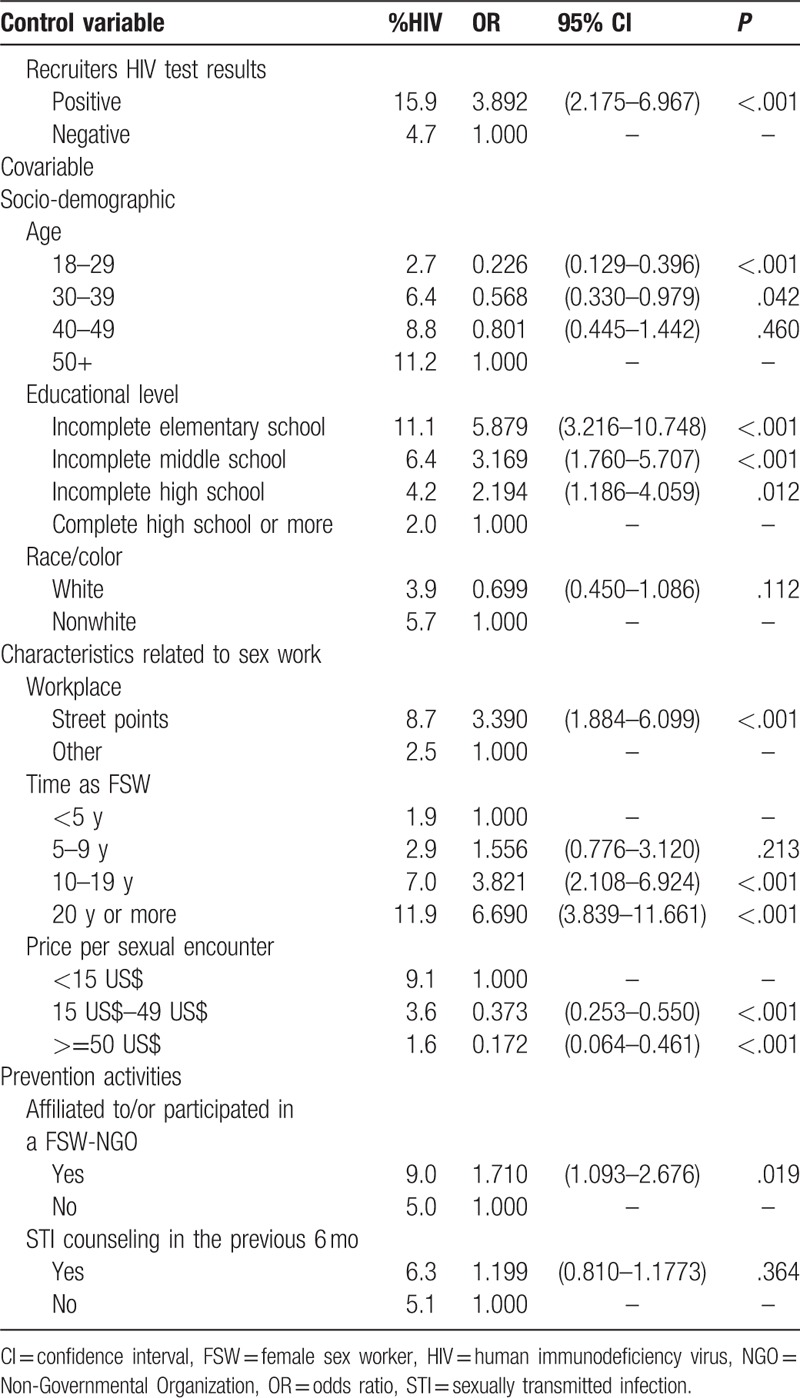
Analysis of the association between socio-demographic variables, characteristics related to sex work, prevention activities and HIV infection adjusted by the recruiter's HIV test results among FSW, Brazil, 2016.

In the logistic regression analyses presented in Table 1, many of the studied variables were significantly associated with HIV infection, even after controlling for HIV recruiter's result. Regarding socio-demographic characteristics, the older the FSW the higher the HIV prevalence; and the lower the educational level the higher the odds of HIV infection. However, no statistically significant difference was estimated for skin color/race. Regarding commercial sex characteristics, HIV infection was associated with time in commercial sex work: HIV prevalence ranged from 1.9% for less than 5 years to 11.9% for greater or equal to 20 years of sex work (OR = 6.7, *P* < .001). Sex work venue was also significantly associated with HIV infection, with an OR of 3.4 (*P* < .001) when point of street is compared to other workplaces. Additionally, an inverse association was found for the price of each sexual encounter, the higher the price the smaller the odds of HIV infection. In relation to participation in prevention activities, women who were affiliated to or participated in an FSW-NGO in the past 6 months had 1.7 times greater chance of being HIV infected. STI counseling in the last 6 months prior to the survey was not statistically significant.

Results related to history of violence, use of alcohol and illicit drugs and sexual behavior are presented in Table 2 . The odds of being HIV infected among FSW who had been exposed to sexual violence at least once in a lifetime was significantly higher (OR = 1.5, *P* = .028). As to alcohol use, only the indicator unprotected sex under the effect of alcohol or drug use at least once a week showed a statistically significant association with HIV infection (OR = 2.0, *P* = .010). On the other hand, the use of illicit drugs at least once a week was highly significant: HIV prevalence varied from 4.5% to 10.6%, OR = 2.5 (*P* < .001), with a marked effect for frequent crack use (OR = 3.6, *P* < .001). Although HIV prevalence was smaller for consistent condom use with clients, the OR was not statistically significant. However, the situation of not using condom for not having one available at the time of the sexual encounter was significantly associated with HIV infection (OR = 1.8, *P* = .016). Other circumstances for not using condom such as “many sexual encounters during the day,” “allergy to condom,” “unconsciousness due to use of alcohol or drugs,” or “any other motive” showed borderline associations as well.

**Table 2:**
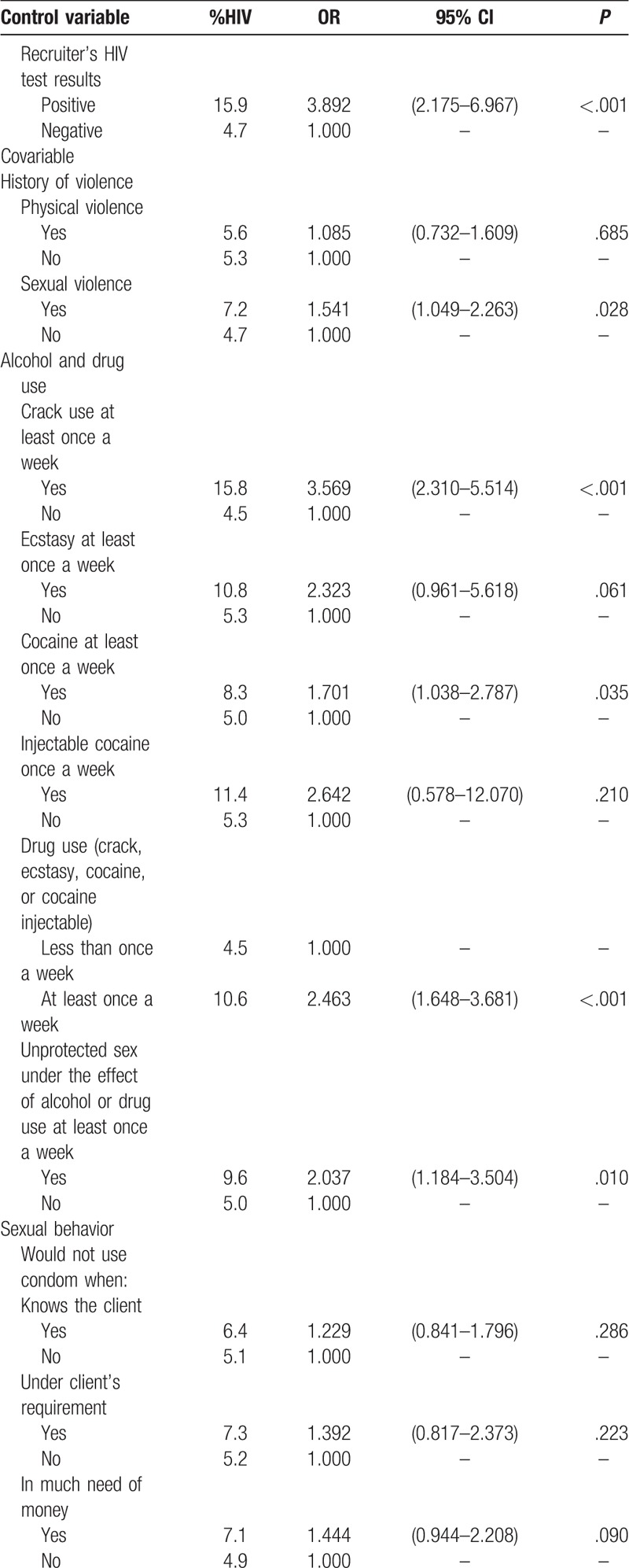
Analysis of the association between history of violence, alcohol and drug use, sexual behavior and HIV infection adjusted by the recruiter's HIV test results among FSW, Brazil, 2016.

**Table 2 (Continued):**
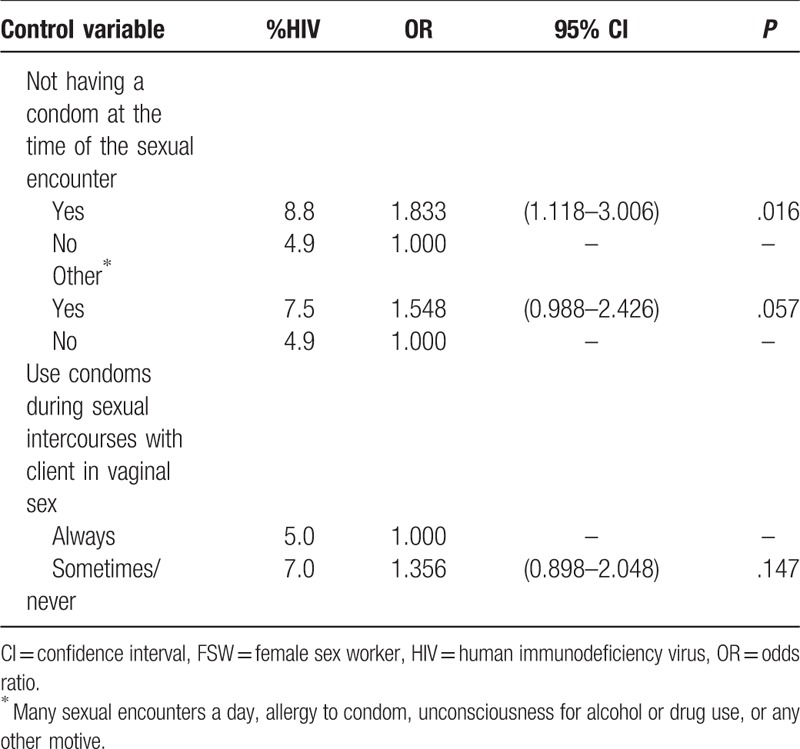
Analysis of the association between history of violence, alcohol and drug use, sexual behavior and HIV infection adjusted by the recruiter's HIV test results among FSW, Brazil, 2016.

Among the indicators of health service utilization, neither uptake of the Pap smear exam nor HIV testing in the previous 24 months before the survey showed a significant effect on HIV infection, although prevalence estimates were smaller among FSW who used health services (Table 3). Regarding the occurrence of STI signs over the 12 months prior to the survey, presence of blisters on the vagina or anus indicated a chance 2.6 times higher of HIV infection when compared to those who did not report STI signs. The OR was highly significant among those FSW who had been exposed to syphilis (OR = 4.6, *P* < .001).

**Table 3:**
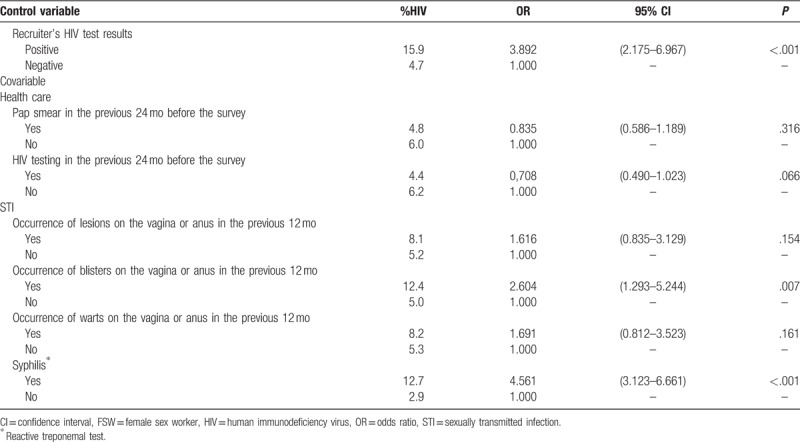
Analysis of the association between utilization of health care, occurrence of STI signs and syphilis^∗^ and HIV infection adjusted by the recruiter's HIV test results among FSW, Brazil, 2016.

In Table 4, we present the results of the multivariate analysis. Educational level remained statistically significant, highlighting the stronger effect of illiteracy or very low level of education, as well as price per sexual encounter, time of exposure to sex work, and the workplace (street vs others) after controlling for all other variables that also showed significant effects on HIV infection. Among the indicators of alcohol and illicit drug use, only the use of crack showed an adjusted significant OR (OR = 1.8, *P* = .027). Syphilis (reactive treponemal test) was the most important predictor of HIV infection, with corresponding adjusted OR of 2.7 (*P* < .001).

**Table 4:**
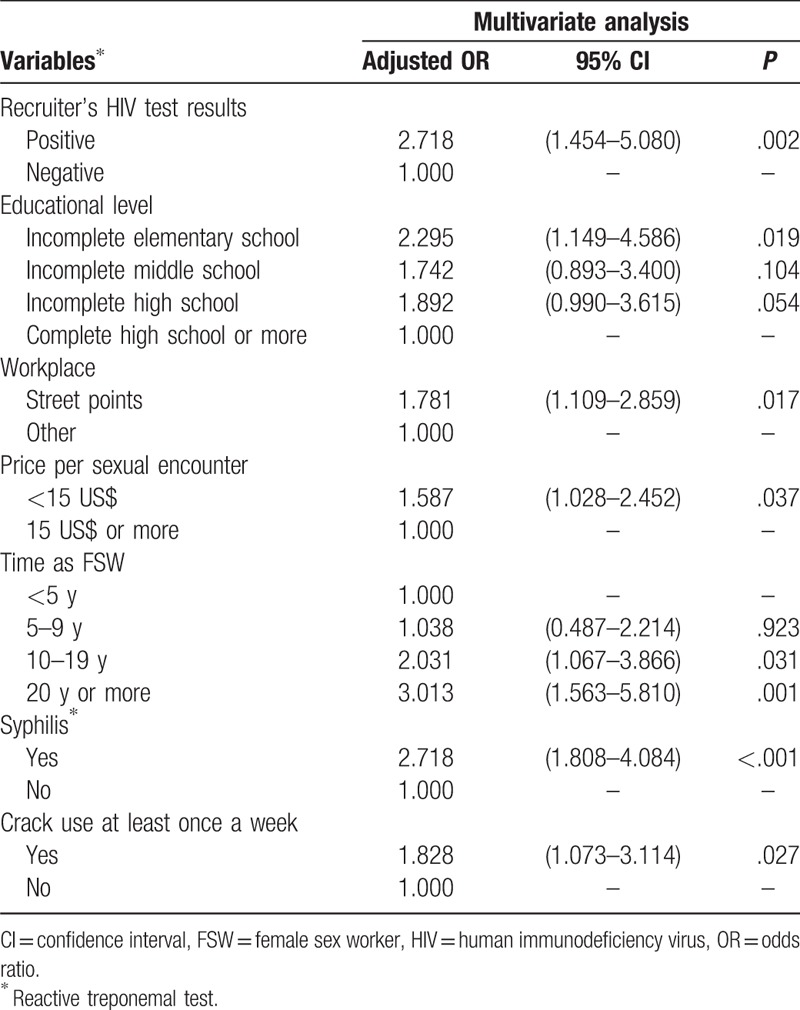
Multivariate analysis of the association between study variables and HIV infection adjusted by the recruiter's HIV test results among FSW, Brazil, 2016.

## 4 Discussion

In the first BSSS among FSW recruited by RDS in 2009 in 10 Brazilian cities,^[38]^ HIV prevalence was estimated as 4.8% (95% CI: 3.4%–6.1%), approximately 12 times higher than the estimated prevalence in the Brazilian female population. Seven years later, the findings of the present study showed no significant change in HIV prevalence, which remain at the same 5% level, with overlapping 95% CI (4.4%–6.2%). A large and significant homophily effect was found as well.

The recruitment of a large number of FSWs in 12 Brazilian cities, in a short time period, at a relatively low cost compared to studies conducted in high-income countries, and the use of appropriate statistical procedures in data analysis, indicate that RDS is a feasible methodology for the study of FSW in Brazil. The experience of the previous RDS study enabled us to improve the techniques for data analysis and all the logistic regression models used in the present study took into account the HIV infection homophily effect and the intraclass correlation between recruited FSW by the same participant.^[39]^

To identify the main predictors of HIV infection, we constructed indicators based on different aspects that characterize the current HIV/AIDS epidemic in Brazil among FSW. In relation to socio-demographic and commercial sex characteristics, low educational level, street as the main work venue, low price per sexual encounter, and longer exposure time as a sex worker were found to be the main predictors of HIV infection.

Our results corroborate the results of other international studies among FSW^[42–44]^ and the results of the previous 2009 BBSS.^[39]^ Older women, in addition to having a longer period of sex work exposure, who charge less for their services, have lower education levels and, for the most part, work in the streets, are factors that have been shown to be associated with HIV infection. As to the use of alcohol and illicit drug use, our findings reiterate the effects of a greater HIV vulnerability associated to unprotected sex.^[45]^

The possibility of not using condoms in some specific situations, such as not having condom available at the time of the sexual encounter, showed a significant effect on HIV infection as well. Data from previous surveys in Brazil evidenced a tendency for FSW to report consistent condom use with clients, especially when interviewed by health staff. However, when questions are asked indirectly, they reveal not using condoms in several circumstances.^[4,9]^

Regarding participation in prevention activities, the results showed a higher chance of HIV infection among women who reported being affiliated to or participating in FSW-oriented NGOs. This finding suggests that HIV-infected women may have sought the support given by NGO activists because of their HIV infection. Unfortunately, in the current situation of weakening of NGOs in Brazil, the role of these institutions has been less and less focused on prevention and health promotion, as had historically occurred in Brazil.^[46]^

The findings on health services utilization indicated a smaller HIV prevalence among FSW tested for HIV over the past 2 years. Frequency of HIV testing represents the individual concern with preventive health care but also self-perception of risk. Despite the nonsignificant OR, the lower chance of being HIV infected among FSW who had tested for HIV over the past 2 years suggests improvements in HIV testing mainly due to prevention attitudes.^[47,48]^

The occurrence of other STI indicated by the presence of blisters on the vagina or anus and syphilis were the most significant determinants of HIV infection. These findings reveal not only exposure in the past to unsafe practices related to STI,^[9]^ but also may reflect the enhancement of STI on HIV transmission.^[49]^

History of sexual violence was shown to be a relevant factor associated with HIV infection. Although prostitution in Brazil is not considered a crime under the National Constitution, FSW constantly experience human rights violations such as physical and sexual violence usually perpetrated by partners, family members, and clients.^[50]^ According to the World Health Organization,^[51]^ violence has a direct impact on the adoption of safe sex practices among FSW. Engagement in violent and unprotected sexual practices, even against their will, reflects the stigma and discrimination suffered by these women, factors that have been shown to be strongly associated with adverse health outcomes.^[52,53]^

The results of the multivariate analysis showed that the association of some variables with HIV infection persisted, such as effect of lower education and cheaper fee for services, working at street spots, longer exposure time of sex work, syphilis, and crack use at least once a week. It is important to note that the use of multivariate models on the data collected by RDS often renders variables that lose statistical significance due to the complex sampling design with over-control of the homophily effect, or to adjustments for confounding. Other limitations are related to the cross-sectional design, for which the analysis of causality is restricted since temporality is not addressed in this type of study.^[54]^

In conclusion, the main factors associated with HIV infection identified in this multivariate analysis characterize a specific type of street-based commercial sex work in Brazil: older women with none or very low degree of instruction, who charge less for the sexual encounter and frequently engage in higher risk sexual behavior. The small fee per sexual encounter is a determinant of the type of client, in general of low socioeconomic status and who are more likely to request unprotected sex.^[27]^ Besides providing prevention knowledge and health promotion, interventions focusing on low-paying sex workers must emphasize the risk associated to unsafe sexual behavior with both clients and steady partners.^[17]^

Ultimately, although the statistical analyses provide valuable information for developing targeted interventions, there is a further need to address other contextual factors. FSWs are exposed to multiple harms including illicit drug use, violence and criminality, exploitation, as well as stigma and discrimination.^[55]^ Thus, comprehensive social interventions must focus on the multiple needs of this vulnerable population, including individual and contextual factors that can influence sexual behavior.

## Acknowledgments

The authors thank the participants of the study and to the local teams that carried out the fieldwork in the 12 cities. The authors also thank the support of STI/HIV/Aids and Viral Hepatitis Department of the Brazilian Minister of Health; additionally, the support of The Brazilian FSW Group: Célia Landmann Szwarcwald, Paulo Roberto Borges de Souza Júnior, Orlando C Ferreira Jr, Giseli Nogueira Damacena, Neide Gravato da Silva, Rita Bacuri, Helena Brigido, Hermelinda Maia Macena, Ana Brito, Inês Dourado, Mark Drew Crosland Guimarães, Wanessa da Silva de Almeida, Alexandre Grangeiro, Carla Luppi, Karin Regina Luhm, Isete Maria Stella, Adriana Varela Espinola, Tânia Varela, and Francisca Sueli da Silva.
